# Perspective on solving the problem of declining interest in physical activity in Poland

**DOI:** 10.3389/fspor.2024.1416154

**Published:** 2024-07-02

**Authors:** Paweł Adam Piepiora, Justyna Bagińska, Zbigniew Norbert Piepiora

**Affiliations:** ^1^ Faculty of Physical Education and Sports, Wroclaw University of Health and Sport Sciences, Wrocław, Poland; ^2^ Faculty of Humanities and Social Studies, Karkonosze University of Applied Sciences in Jelenia Gora, Jelenia Góra, Poland; ^3^ Faculty of Spatial Management and Landscape Architecture, Wroclaw University of Environmental and Life Sciences, Wrocław, Poland

**Keywords:** generation BB, generation X, generation Y, generation Z, generation Alpha

## Abstract

One of the leading areas of Polish research addressed in the physical culture sciences, is the declining interest in physical activity. The likely reason for this situation may be the inadequate communication of physical culture to today's generations: BB (baby boomers), X (great unknowns), Y (millennials), Z (snowflakes), Alpha (digital). Therefore, the aim of this article is to address the problem of declining interest in physical activity in Poland by identifying the appropriate approach of teachers, trainers and instructors to today's generations. The specifics of BB, X, Y, Z and Alpha generations are described and their expectations regarding physical activity are indicated. It was concluded that activating these social groups should be done by prompting topics that are important to them. Thus, BB value organizational stability, X need to see the purposefulness and attractiveness of the activities, Y equate physical activity with personal development, Z only take up useful forms of activities, and finally Alpha like smart technology-assisted activities.

## Introduction

Physical culture is a socially universal phenomenon (1). It refers to physical activity in its broadest sense in the areas of physical education, rehabilitation, recreation, sport (amateur, competitive, professional), and tourism (2). It emphasizes the impact of physical activity on the physical and mental health of society, which is documented by numerous studies that concern the formation of correct bodily habits and attitudes, personality, self-discipline and functioning in society (3).

For people at different periods of life, slightly different goals for undertaking physical activity are noted. In adolescence, it is the need to satisfy exercise (4). In the teenage period, it is the need for competition and comparison (5). While in adulthood it is the need to maintain physical fitness and beauty (6). And in late adulthood, it is the need to delay the aging process and injury (7).

But a Polish problem that is still relevant in the physical culture sciences is the decline in interest in physical activity, especially among younger generations (8). Analysing generational changes in Poland after the Second World War, a downward trend in taking up physical activity was noted (9). The reason for this may lie in the inadequate transmission of physical culture to contemporary generations: BB (baby boomers), X (great unknowns), Y (millennials), Z (snowflakes), and Alpha (digital) (10). These generations function in parallel. Despite intergenerational differences, some commonalities are noticeable, which shape social relations (11). First, each generation can use technology, but to a different extent. Moreover, they all want to change the world for the better and want to be happy. But what unites the generations divides them at the same time into perspectives on their understanding of technology, the world and happiness through generationally individualised values.

In view of the above, the aim of this article is to present a perspective for solving the declining interest in physical activity in Poland by identifying the appropriate approach of physical culture professionals to representatives of contemporary generations. This must refer to understanding the reasoning of BB, X, Y, Z and Alpha's, involvement in physical activity. This is why it is so important for the physical culture sciences to know about the contemporary generations, which are marked by technological progress. To collaborate effectively with them, it is first necessary to know their characteristics.

## Characteristics of today’s generations

### Generation BB (baby boomers)

People who were born in Poland during the large baby boom between 1946 and 1964 are referred to as the BB generation, or baby boomers. The youngest BBs are in their sixties and are still economically active (12). They have spent years independently building their high positions in their local communities and are, to a greater or lesser extent, authorities for the younger generations. The BBs identify success with systematic work. They take traditional values as appropriate and prefer a conservative family model. They regard reaching retirement as a stage of life that can be devoted to hobbies (13). They do not like change, a sense of stability is important to them, so they are persistent in each job and their resolutions. Representatives of this generation are loyal and respect authority figures, while feeling at their best when they know they are really needed (14).

The BBs like to engage in those physical activities that suit them. Moreover, they are systematic and determined in their exercise. They correctly see physical activity as a means of maintaining physical and mental health. But it should be noted that sudden changes cannot be made in BB activity programmes. For example: an increase in the payment for exercise classes cannot take place from month to month. An analogous situation applies to certain assumptions of the class structure. Any changes to the class programmes for the BB are acceptable to them after the season (half-year or a year) is over. This is the only way to keep them engaged in a specific physical activity. They lose motivation when the original arrangements for organising physical activities are changed unexpectedly.

### Generation X (great unknown)

People who were born between 1965 and 1980 are referred to as generation X, the great unknown. The youngest X are 44 years old and active in the labour market (15). The name of this generation is related to a period of uncertain times, where, on the one hand, everything was subordinated to the People's Republic of Poland and, on the other hand, society opposed this order. Therefore, the X do not divide society by wealth, but value high quality and leisure time. Tradition is also especially important to them. They like peace and quiet around them and are usually not conflictual (16). They are loyal to one job if they are fairly compensated. And this manifests itself in their efficiency, specific work experiences and social sensitivity (17).

The X engage in physical activity if they have a goal, and its form is attractive to them. They want to feel pleasure from spending time on bodily exercise. For example: among women, there is a growing interest in contemporary forms of dance and fitness, and among men, athletic forms of movement and team sports games predominate. Therefore, medical indications for physical activity are not underestimated by the X. The problem is finding interesting forms of activities for them in the vicinity. This is particularly evident in small towns. But once the X engage in physical activity, they are determined and expect occasional rewards from the instructor in the form of praise and feedback on their progress.

### Generation Y (millennials)

People who were born between 1981 and 1995 are referred to as Generation Y, or millennials. This is the last generation of the 20th century. The youngest Y are 29 years old and are swift in the labour market (18). They value self-employment and free time just for themselves. But personal development is equally important to them. Therefore, changing jobs is not a problem for the Y. They start multiple tasks at the same time, but constantly change their priority (19). The Y like challenges and a relaxed atmosphere at work, but because of their lifestyle they are often perceived as reckless, disloyal and capricious. To keep the Y motivated in anything, they need to be constantly praised. If they receive praise and do what they like, they are good and fulfilled employees (20).

If the Y come to the first class, it means that they are already determined. They value different forms of physical activity that coincide with their plans for personal development. Therefore, engaged and motivated by the instructor in the exercises, they explore the form of physical activity in detail. For example: millennials training in karate take an in-depth interest in the development of the discipline, the philosophy, the ranking of athletes, etc. It is relevant that the Y will stop engaging in a particular physical activity or change club overnight if they find that they are no longer thriving there. In such cases, the Y should not be stopped, as this can only project a negative opinion of a particular instructor in their eyes.

### Generation Z (snowflakes)

People who were born in Poland between 1996 and 2010 are referred to as Generation Z, or snowflakes. This is the generation from the turn of the 20th and 21st century, which was mostly brought up stress-free. The Z got what they wanted and were pampered, making most of them hard to manage stress and failure. They are subtle and delicate, hence the name snowflakes. The youngest Zs are 14 years old and the oldest have just entered the job market (21). They are technologically proficient, value internet access and like to work remotely, which enables them to travel frequently (22). For the Z, it is the norm to do several things at the same time, but for the same reason they are unable to focus on just one course of action. Therefore, they succumb to trends. They are characterised by realism, creativity, personal development and independence. The Z are quick to judge given phenomena, are pessimistic and critical. They identify the desirability of work only with personal development, thus constantly change it (23).

They take up only those forms of physical activity that are useful to them and related to their career. More often than not, they take up a sport to acquire new skills. It is important to note that when the Z feel strong in a particular activity, they immediately switch to another one. Unlike the Y, they naturally switch from one physical activity to another. The norm for them is to train successively in any combat sport, team sports game, swimming, MTB, athletics, etc. Therefore, the Z should not be held back in their decisions but should be shown the next physical activities that are significant for their progressive functioning.

### Generation Alpha (digital)

Those born between 2011 and 2025 are referred to as the Alpha, or digital, generation. This is the first generation born entirely in the 21st century. The oldest Alphas are 13 years old, and the youngest will be born by the end of 2025 (24). They do not know a world without the internet and a life without constant use of modern technologies. That is why they are called digital. They like to learn and play on apps (25). Because of this, they find it difficult to function offline. This is noticeable in their short attention span, their lack of initiative in organising their leisure time and their difficulty in establishing relationships (26). At the same time, they hate boredom and like their parents to initiate activities for them. Therefore, Alphas, using smart technology naturally, are eager to undertake a variety of physical activities that are set by online trends. But enforcing regularity and motivating them to participate in these activities falls on the parents.

Alphas’ personal and purposeful search for activity is geared towards e-sports or popcorn gaming. It is worth noting that, once an Alpha has come to a physical activity class, it is because of their legal guardians. Therefore, the perception of the physical activity in question by both is important. Alphas need to be constantly motivated and like to constantly monitor (as in digital games) the effects of their exercise. Therefore, Alphas’ use of smartwatches in physical activities is fully justified. But it should be stressed that if an activity is considered boring by an Alpha, they will immediately abandon it and their parents will seek other physical activities for their kids that are promoted online.

## Discussion

This article characterises contemporary generations of Poles in relation to the physical culture sciences. It describes what is important to them in undertaking physical activity. It was concluded that activating Polish society to physical activity should be done by influencing issues relevant to the living generations: BB, X, Y, Z and Alpha. Efforts should be made to learn about the characteristics of these generations. A good knowledge of the expectations of the representatives of the different generations will probably contribute to greater effectiveness of the physical activities undertaken by them. In turn, the positive effects of these exercise activities will translate into increased feelings of satisfaction among them. This will translate into a favourable opinion of the teacher, trainer or instructor. Therefore, the view is taken that high ratings for activities tailored to each generation can provide a solution to the problem of declining interest in physical activity in Poland.

It is also important to hold classes for intergenerational groups. In such cases, an individual approach to representatives of different generations and firm implementation of the established exercise programme are of great importance. The sporting experience of older generations can be effectively passed on to younger generations with the rational use of smart technology. This form of intergenerational communication can be an attractive enhancement of mutual learning (27).

Therefore, the following viewpoint is adopted: the social engagement of Poles in physical activity can be increased by combining intergenerational relationships based on existing resources, understanding, communication and openness to technological innovations (11). In this sense, older generations are responsible for educating younger generations. But technological innovations cannot be prioritised over long-established educational methods (28). Common sense needs to be exercised in all of this, as, for example, an excessive focus on smart technology does not shape important social skills in young people. For instance, young people in cyberspace are constantly being exposed to the escalation of the pathology of sport by the contemporary fashion of promoting mixed martial arts, which cannot be considered a sport (29). Firstly, in mixed martial arts the right to necessary defence is notoriously violated. Secondly, massacring a human being in competition is not in line with the White Paper on Sport (30). Thirdly, there is no country that recognises mixed martial arts as a sport. In such cases, the wisdom and experience of older generations are invaluable (31). It is thanks to them that we know that the correct term for mixed martial arts is neogladiatorism, which camouflages itself under the banner of martial arts (32). This kind of information is not widely available in cyberspace for younger generations. Therefore, the example described above highlights the role of physical culture specialists in Polish society in educating the next generations.

### Limitations of perspective

It should be stressed that this perspective does not consider the problems of the demographic decline and the associated indulgence and excessive focus of parents on the welfare of their children.

### Practical recommendations

Teachers, trainers and instructors alike need to have an appropriate pedagogical background, including knowledge of the present-day generations. Therefore, regardless of which generation they come from themselves, they should have the pedagogical skills to work with the members of BB, X, Y, Z, and Alpha generations in order to achieve high effectiveness of the implemented physical activity processes.

## Conclusions

Nowadays in Poland, to conduct effective classes in any physical activity, it is necessary to approach representatives of the contemporary generations in accordance with their expectations. The BB are eager to get active in classes, but do not like sudden changes to the original arrangements; the X are emboldened to exercise if they have a specific goal and the form of the activity is attractive to them; the Y activate to activities when they are identified strictly with personal development; the Z only take up physical activities that they feel are useful. Finally, the Alphas are engaged in physical activities by their parents, but the effectiveness of these activities depends on the use of smart technology. An appropriate approach to these generations may therefore be the key to solving the problem of declining interest in physical activity in Poland, leaving aside the demographic decline.

## Data Availability

The original contributions presented in the study are included in the article/Supplementary Material, further inquiries can be directed to the corresponding author.
